# MoGCN: A Multi-Omics Integration Method Based on Graph Convolutional Network for Cancer Subtype Analysis

**DOI:** 10.3389/fgene.2022.806842

**Published:** 2022-02-02

**Authors:** Xiao Li, Jie Ma, Ling Leng, Mingfei Han, Mansheng Li, Fuchu He, Yunping Zhu

**Affiliations:** ^1^ State Key Laboratory of Proteomics, Beijing Proteome Research Center, National Center for Protein Sciences (Beijing), Beijing Institute of Life Omics, Beijing, China; ^2^ Stem Cell and Regenerative Medicine Lab, Department of Medical Science Research Center, State Key Laboratory of Complex Severe and Rare Diseases, Translational Medicine Center, Peking Union Medical College Hospital, Peking Union Medical College and Chinese Academy of Medical Sciences, Beijing, China

**Keywords:** multi-omics integration, graph convolutional network, autoencoder, similarity network fusion, cancer subtype classification

## Abstract

In light of the rapid accumulation of large-scale omics datasets, numerous studies have attempted to characterize the molecular and clinical features of cancers from a multi-omics perspective. However, there are great challenges in integrating multi-omics using machine learning methods for cancer subtype classification. In this study, MoGCN, a multi-omics integration model based on graph convolutional network (GCN) was developed for cancer subtype classification and analysis. Genomics, transcriptomics and proteomics datasets for 511 breast invasive carcinoma (BRCA) samples were downloaded from the Cancer Genome Atlas (TCGA). The autoencoder (AE) and the similarity network fusion (SNF) methods were used to reduce dimensionality and construct the patient similarity network (PSN), respectively. Then the vector features and the PSN were input into the GCN for training and testing. Feature extraction and network visualization were used for further biological knowledge discovery and subtype classification. In the analysis of multi-dimensional omics data of the BRCA samples in TCGA, MoGCN achieved the highest accuracy in cancer subtype classification compared with several popular algorithms. Moreover, MoGCN can extract the most significant features of each omics layer and provide candidate functional molecules for further analysis of their biological effects. And network visualization showed that MoGCN could make clinically intuitive diagnosis. The generality of MoGCN was proven on the TCGA pan-kidney cancer datasets. MoGCN and datasets are public available at https://github.com/Lifoof/MoGCN. Our study shows that MoGCN performs well for heterogeneous data integration and the interpretability of classification results, which confers great potential for applications in biomarker identification and clinical diagnosis.

## 1 Introduction

Owing to the recent rapid developments in high-throughput sequencing technology, multi-omics research has strongly promoted the development of precision medicine. However, the application of precision medicine for the prevention, diagnosis, and treatment of tumors is far from satisfactory (Lu and Zhan, 2018). Multi-omics approaches are novel frameworks that can integrate multiple omics datasets generated from the same patients (Heo et al., 2021); thus, an increasing number of studies have tried to characterize the molecular and clinical features of cancers from a multi-omics perspective (Sun and Hu, 2016).

Integrated multi-omics approaches can be divided into two types: the integration of Euclidean structure data or the integration of non-Euclidean structure data (Eicher et al., 2020). The first approach uses the expression matrix as the input, and then trains machine learning models for clustering and classification. For example, Chaudhary et al. were the first to use a deep autoencoder (AE) (Hinton and Salakhutdinov, 2006) model to predict the survival of patients with hepatocellular carcinoma (Chaudhary et al., 2018); Chen et al. designed a deep-learning framework, DeepType, that performs a joint model of supervised classification, unsupervised clustering, and dimensionality reduction to learn cancer-relevant data representation (Chen et al., 2020). These methods can handle large-scale datasets, but require substantial effort to interpret how specific features contribute to the predicted results. On the other hand, the non-Euclidean data integration approach trains models using the network topology data. These methods can identify cancer subtypes by fusing the similarities derived from various omics data, such as similarity network fusion (SNF) (Wang et al., 2014), GrassmannCluster (Ding et al., 2019), and high-order path elucidated similarity (HOPES) (Xu et al., 2019). These network-based processes are clinically intuitive, but existing studies have focused on the unsupervised integration of multi-omics datasets.

Meanwhile, classification of tumor subtypes plays a leading role in the treatment and prognosis of cancers. This is a multi-class classification task and has always presented a challenge in the field of integrating multi-omics using machine learning. There is an urgent need for a multi-class network classification model to handle cancer subtype classification and biomarker identification. Graph convolutional network (GCN) (Kipf and Welling, 2017) is a recently developed approach to incorporate graph structures into a deep learning framework. It classifies unlabeled nodes using information from the topology of the network as well as the feature vectors of the nodes. The network structure makes GCN naturally interpretable. Several studies have been reported to use this model to predict the complex genome-disease association (Xuan et al., 2019) and drug-disease associations (Liu et al., 2020; Yu et al., 2021).

Herein, we developed MoGCN, a multi-omics integration model based on graph convolutional network, for cancer subtype analysis. This study creatively proposes developing a network diagnosis model based on the pipeline of “integrating multi-omics data first and then performing classification”. Specifically, we utilized AE to integrate multi-omics expression data and SNF to integrate a typical network topology data patient similarity network (PSN) (Pai and Bader, 2018), to construct a comprehensive view of cancer patients. Then, we used GCN to combine the AE and SNF results and construct the final model for cancer subtype classification. By applying MoGCN on the breast invasive carcinoma (BRCA) data in The Cancer Genome Atlas (TCGA, http://cancergenome.nih.gov/), we demonstrated that MoGCN could achieve the best performance in cancer subtype classification among the current algorithms. Similarly, MoGCN achieved good results on the TCGA pan-kidney cancer validation dataset. The case study for breast cancer also shows that our method has great potential for heterogeneous data integration, marker identification, and clinical diagnosis.

## 2 Materials and Methods

**FIGURE 1:**
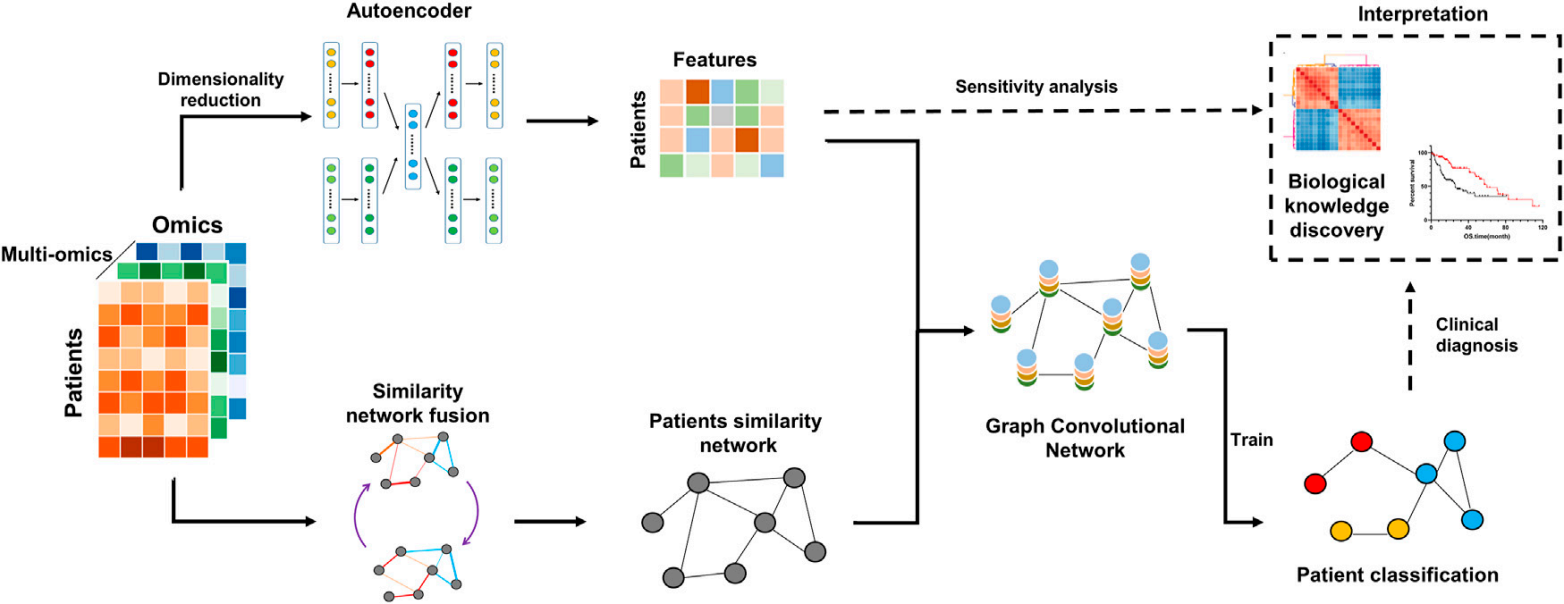
MoGCN workflow schematic. The input is the multi-omics data. First, the AE and SNF methods are used to reduce dimensionality and to construct the patient similarity network, respectively. Next, the vector features and adjacency matrix are fed into the GCN for training. Feature extraction and network visualization can be used for further biological knowledge discovery.

MoGCN uses multi-omics expression datasets from patients as inputs, including but not limited to genomics, transcriptomics, and proteomics datasets. First, we applied the autoencoder model to extract patient expression features (expression matrix), and applied the similarity network fusion model to construct a patient similarity network. Then, we used the GCN model to integrate these two types of heterogeneous features and to train the cancer subtype classification model. By integrating network and vector characteristics, MoGCN was able to achieve good classification performance, and effectively addressed the issue of deep learning interpretability in clinical applications. MoGCN is a command-line tool that allows users to integrate multi-omics datasets for cancer subtyping classifications efficiently. The overall workflow is shown in Figure 1.

### 2.1 Data Preparation

BRCA datasets were downloaded from the UCSC Xena browser (https://xenabrowser.net/) and the Cancer Proteome Atlas (TCPA) portal (https://tcpaportal.org/tcpa/) and processed. Copy number variation (CNV) data at the genomic level, RNA-seq data at the transcriptomic level, reverse phase protein array data (RPPA) at the proteomic level, and clinical data were all available. The breast tumors were classified into four subtypes (Cancer Genome Atlas Network, 2012): Basal-like, typically with no expres-sion of hormone receptors or ERBB2; Her2-enriched, overexpressing the oncogene ERBB2; and Luminal A and B, generally estrogen receptor (ER)-positive tumors expressing epithelial markers (Luminal B shows a higher Ki67 index and worse prognosis than Luminal A); these were similar to results generated by the established and widely used PAM50 assay. Common samples were collected from each omics level; therefore, data from a total of 511 patients in the BRCA dataset were obtained. The details of the dataset are shown in Table 1.

**TABLE 1:** Summary of the BRCA dataset.

Number of samples		Number of features	
Basal-like	112	CNV	19,273
Her2-enriched	53	mRNA	19,580
Luminal A	248	RPPA	223
Luminal B	98	—	—
Total	511	Total	39,076

A 10-fold cross validation method was applied to all algorithms implemented in this study. The dataset of 511 samples was first divided randomly into 10 subsets. We successively selected one subset to become a testing dataset, while the others were used as a training dataset. Therefore, 10 combinations of the training dataset and testing dataset were obtained. In each run, we used the training dataset to train the model and the testing dataset to test the model’s performance; the average result of the 10 runs was taken as the final result of the model.

### 2.2 Multi-Modal Autoencoder

The autoencoder consists of two modules simultaneously: an encoder and a decoder. The encoder (*f*) maps the original domain *X* to a new domain named latent space *Z* with dimension *L*. Then, the decoder (*g*) maps *Z* back to the original input space *X*. The encoder and decoder are defined as *z = f (x)* and $\tilde{x}$ *= g (z).* By minimizing the reconstruction loss, the model captures the significant features of the data. The loss function to minimize is formalized as: *E = argmin*_*f,g*_ *[Loss (x, g (f (x)))].*

As the input data are characterized by multi-omics data types and represented by multiple matrices *X*_*1*_ *, X*_*2*_ *, X*_*n*_, corresponding to the genome, transcriptome, proteome matrices, and so on, the autoencoder must have multiple inputs and outputs. A multi-modal autoencoder architecture is proposed. As shown in Figure 1, the model consists of multiple encoders and decoders, which share the same latent layer. The loss function to minimize is formalized as:

$$E={\mathrm{argmin}}_{\mathrm{f,g}}\left(\alpha {\mathrm{Loss}}_{1}\left(x1\mathrm{,g}1\left(f1\left(x1\right)\right)\right)+\ldots +\beta Loss_{2}\left(x1\mathrm{,g}1\left(f1\left(x1\right)\right)\right)\right)$$ (1)

Where *α, … , β* are the weights (prior knowledge) of each data type, and *α + … + β = 1*.

### 2.3 Similarity Network Fusion

The SNF algorithm integrates different types of omics data, creating a network for each data type, and ultimately establishing a comprehensive view of the disease or biological process. SNF is able to compute and fuse PSNs for each data type, which enable the exploitation of complementary information from multi-omics data types and outperforms other single data analysis methods. Specifically, the algorithm computes patient-patient similarity matrices for each data type and constructs patient-patient similarity networks. Then, network fusion is performed to enhance strong connections and remove weak connections. Finally, a fused patient similarity network is established.

Based on the assumption that there were *n* samples (such as patients) and *m* different data types, for the *v*_*th*_ (*v* = 1, 2, …, *m*) data type, a scaled exponential similarity matrix was calculated:

$$W\left(i,j\right)=exp\left(-\frac{\rho^{2}\;\left(x_{i},x_{j}\right)}{\mu \varepsilon_{i,j}}\right)$$ (2)

ρ(i,j) represents the Euclidean distance between the patient x_i_ and x_j_ W(i,j) represents the *n × n* similarity matrix between patient x_i_ and x_j_ *µ* is a hyperparameter that can be empirically set, and *ε* is used to eliminate the scaling problem. Then, the similarity matrix *P* ^*(v)*^ of all patients and K-nearest similarity matrix *S* ^*(v)*^ can be defined as

$$P\left(i,j\right)=\{\begin{array}{c} \frac{W\left(i,j\right)\;}{2\sum_{k\neq i}W\;\left(i,k\right)},\;j\neq i\\ \frac{1}{2},j=i \end{array},S\left(i,j\right)=\{\begin{array}{c} \frac{W\left(i,j\right)\;}{\sum_{k\in N_{i}}W\left(i,k\right)\;},\;j\in N_{i}\\ 0,\;otherwise \end{array}$$ (3)

Then, for the case in which there was two types of data, the process was as follows:

a Calculate $P^{\left(1\right)}$ , $P^{\left(2\right)}$ , $S^{\left(1\right)}$ , $S^{\left(2\right)}$ . Let $P_{t=0}^{\left(1\right)}$ = $P^{\left(1\right)}$ and $P_{t=0}^{\left(2\right)}$ = $P^{\left(2\right)}$ represent the initial two status matrices at *t = 0*.
b Iteratively update the similarity matrix.

$$P_{t+1}^{\left(1\right)}=\;S^{\left(1\right)}\times P_{t}^{\left(2\right)}\times {\left(S^{\left(1\right)}\right)}^{T},P_{t+1}^{\left(2\right)}=\;S^{\left(2\right)}\times P_{t}^{\left(1\right)}\times {\left(S^{\left(2\right)}\right)}^{T}$$ (4)

c After *t* steps, the overall state matrix can be calculated by:

$$\;P^{\left(t\right)}=\;\frac{P_{t}^{\left(1\right)}+P_{t}^{\left(2\right)}}{2}$$ (5)

For the generalization of *m* > 2, the update process is:

$$\;\;\;\;\;\;\;\;P^{\left(v\right)}=\;S^{\left(v\right)}\times \left(\frac{\sum_{k\neq v}P^{\left(k\right)}}{m-1}\right)\times {\left(S^{\left(v\right)}\right)}^{T},\;v=1,2,\;\ldots ,\;m$$ (6)

### 2.4 Graph Convolutional Network

GCN analysis requires two inputs: the structure of the graph and the features of each node. In this manual, one input is the multi-omics feature matrix *X ∈ R* ^*n×d*^, where *n* is the number of nodes and *d* is the number of features. Another input is the PSN, which can be represented by the form of an adjacency matrix *A* ∈ *R* ^*n×n*^. The GCN is built by stacking multiple convolutional layers. Specifically, each layer is defined as:

$$H^{\left(1+1\right)}=\sigma \left({LH}^{\left(I\right)}W^{\left(I\right)}\right)$$ (7)

Where $L={\tilde{D}}^{-\frac{1}{2}}\tilde{A}{\tilde{D}}^{-\frac{1}{2}}$ or $L={\tilde{D}}^{-1}\tilde{A}$ denotes the normalized graph laplacian; $\tilde{A\;}=\;A+I$ denotes the adjacency matrix with added self-connections; $\tilde{D}$ is the degree matrix of $\tilde{A}$ ; *W* is the weight matrix learned from training; *σ* denotes the nonlinear activation function, generally the *ReLU* activation function; and *H* ^*(l)*^ is the input of each layer, and notably, *H* ^*(0)*^ *= X.*

### 2.5 Interpretability of MoGCN

Machine learning has great potential for improving products, processes, and research. However, computers usually do not explain their predictions, which is a barrier to the adoption of machine learning. In this study, the interpretability of MoGCN is reflected in both AE feature extraction and PSN visualization. In the autoencoder model, we used sensitivity analysis (Garson, 1991) for feature extraction: 1) multiplying the standard deviation of each input node by its connection weights in the network; 2) extracting top features every 10 epochs; and 3) merging and sorting the extracted features. The weights analysis method allows feature extraction during the training process, but consumes relatively little extra time. Meanwhile, the visualization of the PSN also provides an intuitive explanation for the clinical diagnosis of patient subtyping.

Sensitivity analysis is a valuable method used to describe the importance of input variables in neural networks quantitatively. The importance of a node can be determined by the variance of this feature (also known as variable sensitivity) and the weighted connections that the node contributes to the network (also known as weight sensitivity). Therefore, the importance score of a feature *x*_*i*_ can be defined as:

$$S_{i}=\sigma_{i}\times \overset{L}{\underset{j=1}{\sum }}\left|W_{ij}\right|$$ (8)

Where *σ*_*i*_ represents the standard deviation of *x*_*i*_, *L* is the number of nodes in the next layer, and W is the connection weight of the input nodes to the output nodes.

In order to obtain stable characteristics of AE during training, the process for each omics layer is as follows:

a Calculate *Si* and extract top *N* features every 10 epochs to get feature sets G_1_, G_2_, …, G_m_.
b After training, merge G_1_, G_2_, …, G_m_ and obtain the stable set of essential genes.

The case study on breast cancer demonstrates the promising potential of MoGCN in biological knowledge mining.

### 2.6 Mainstream Feature Extraction Methods and Classification Methods

We compared AE with the following unsupervised feature extraction algorithms: principal component analysis (PCA), factor analysis (FA), independent component analysis (ICA), and singular value decomposition (SVD). These methods were implemented by calling the built-in functions in the Python scikit-learn library (https://scikit-learn.org/stable/).

We compared GCN with the following state-of-the-art methods: decision tree (DT), K-nearest neighbors (KNN), Gaussian naïve Bayes (GNB), random forests (RF), support vector machine (SVM), deep neural network (DNN) with four layers, GrassmannCluster and HOPES. GrassmannCluster and HOPES were implemented using Matlab. Moreover, other methods were also implemented by calling the built-in functions in the Python scikit-learn library (https://scikit-learn.org/stable/).

### 2.7 Evaluation Index of Model Performance

In the classification tasks, the prediction results of a model have four basic indicators: true positive (TP), false positive (FP), false negative (FN), and true negative (TN). The accuracy represents the proportion of all samples judged correctly by the classifier, and is defined as:

$$accuracy=\frac{TP+TN}{TP+FP+TN+FN}$$ (9)

The *F1* score is a measure of classification tasks. It is often used as the final evaluation index in most machine learning competitions. It is the harmonic average of the precision rate and the recall rate, which has a maximum of 1 and a minimum of 0. It is defined as:

$$F1\;score=2\times \frac{precision\times recall}{precision+recall}$$ (10)

Where *precision* = $\frac{TP}{TP+FP}$ , *recall* = $\frac{TP}{TP+FN}$ .

In addition, all results were subjected to 10-fold cross validation.

### 2.8 Functional Enrichment Analysis

Biological Process (BP) annotation, Molecular Function (MF) annotation and Kyoto Encyclopedia of Genes and Genomes (KEGG) pathway enrichment analyses for selected genes were conducted using David (https://david.ncifcrf.gov/). Gene set variation analysis (GSVA) (Hanzelmann et al., 2013) was performed on the MSigDB (https://www.gsea-msigdb.org/gsea/msigdb/) “c2. cp.reactome.v7.4. symbols.gmt” gene set using the “GSVA” package in R software. *p*-value of <0.05 was considered statistically significant.

### 2.9 Kaplan-Meier Survival Analysis

We used the validation breast cancer cohort (*n* = 1880) from Kaplan–Meier (KM) plotter (https://kmplot.com/analysis/) to validate the prognostic value of genes. 10-years overall survival analysis was performed.

## 3 Results

### 3.1 Multi-Omics Integration Using AE Can Improve Classification Performance

Multi-omics data sets are inherently high-dimensional, and their processing may be computationally intensive. Dimensionality reduction is a general strategy to reduce computational burden. Moreover, multi-omics data are highly heterogeneous and the relationship between different data types (also named as layers in data form) is not linear. The extraction of important features from the various layers remains a huge challenge. Here, we used random forest as a benchmark classifier to compare the performance of different dimensionality reduction algorithms (Tables 2, 3). The results in the rows show that AE performs best in most cases. More importantly, the results in the columns show that after the integration of different omics features, the performance of AE-based classification improved, whereas that of other methods slightly decreased or remained unchanged. The potential reasons for this were: 1) a large amount of noise in multi-omics data, so the classification relative information density is low, which interferes with the traditional algorithms; 2) traditional algorithms are linear methods and cannot uncover potential nonlinear relationships within complex biological data.

**TABLE 2:** The accuracy of different dimensionality reduction algorithms.

	PCA	FA	ICA	SVD	AE
mRNA	0.8318 ± 0.0427	0.7808 ± 0.0351	0.6889 ± 0.0301	0.8278 ± 0.0380	0.8357 ± 0.0396
CNV	0.6008 ± 0.0417	0.5949 ± 0.0263	0.5030 ± 0.0339	0.6047 ± 0.0488	0.5695 ± 0.0497
RPPA	0.7730 ± 0.0199	0.7495 ± 0.0411	0.5440 ± 0.0294	0.7847 ± 0.0474	0.8082 ± 0.0438
mRNA + CNV + RPPA	0.8258 ± 0.0459	0.7044 ± 0.0440	0.6283 ± 0.0441	0.8337 ± 0.0402	0.8787 ± 0.0477

*10-fold cross validation (mean ± standard deviation).

**TABLE 3:** The F1 score of different dimensionality reduction algorithms.

	PCA	FA	ICA	SVD	AE
mRNA	0.8086 ± 0.0534	0.7499 ± 0.0450	0.6226 ± 0.0324	0.8129 ± 0.0428	0.8144 ± 0.0520
CNV	0.5578 ± 0.0504	0.5493 ± 0.0231	0.4295 ± 0.0410	0.5461 ± 0.0413	0.5209 ± 0.0548
RPPA	0.7313 ± 0.0388	0.7098 ± 0.0547	0.4430 ± 0.0438	0.7498 ± 0.0573	0.7935 ± 0.0489
mRNA + CNV + RPPA	0.8080 ± 0.0490	0.6541 ± 0.0489	0.5670 ± 0.0542	0.8172 ± 0.0493	0.8722 ± 0.0529

*10-fold cross validation (mean ± standard deviation).

### 3.2 Integration of PSN for Greater Performance Improvement

**FIGURE 2:**
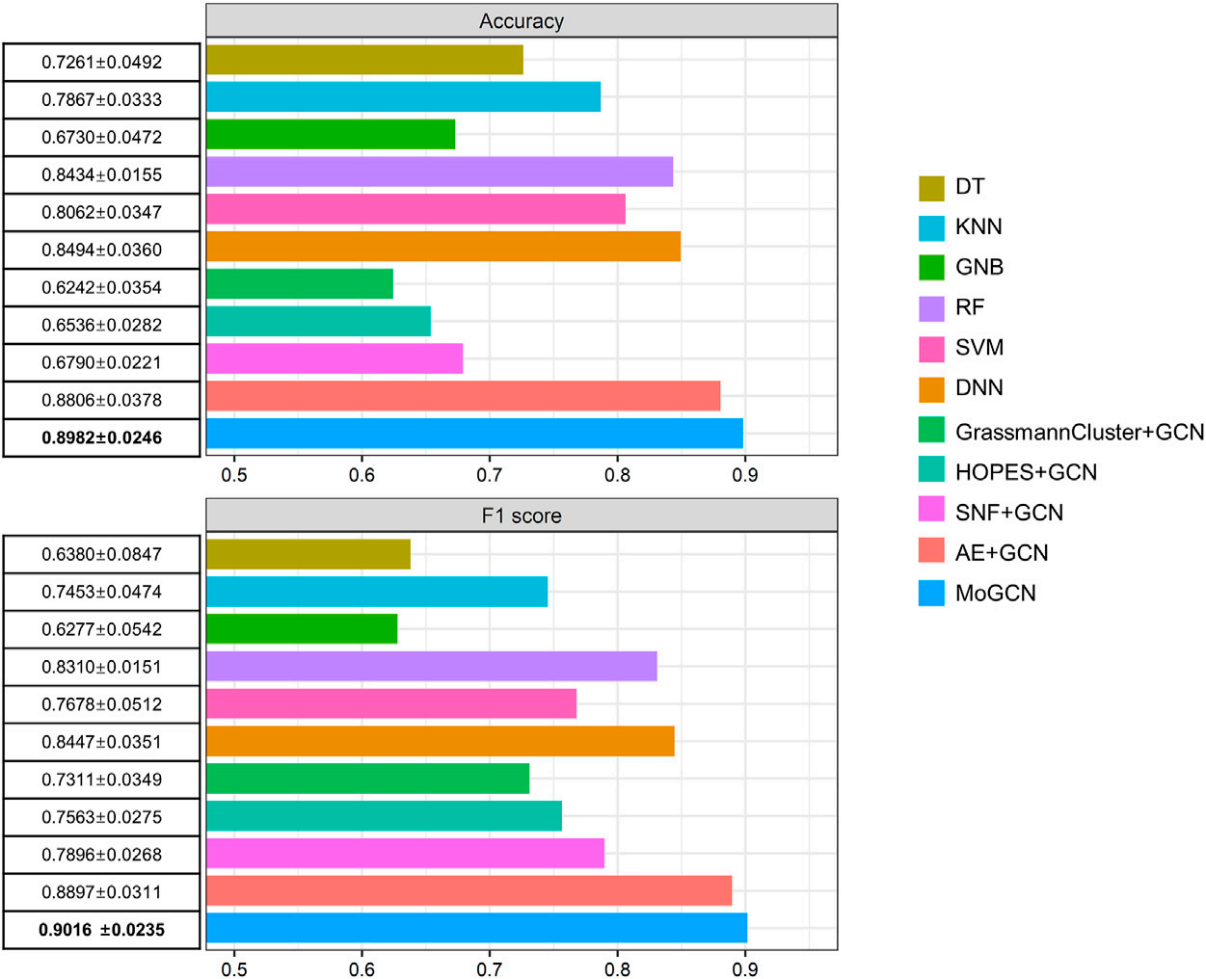
Performance comparison of different algorithms. 10-fold cross validation (mean ± standard deviation).

After integration of multi-omics data using the AE, we applied the SNF model to construct the patient similarity network (PSN) (Figure 1). Then, we used GCN to integrate the expression data and PSNs to establish a complete pipeline for multi-omics biological data. We compared MoGCN with DT, KNN, GNB, RF, SVM, DNN, GrassmannCluster, and HOPES. Considering that GrassmannCluster and HOPES are algorithms used to construct PSNs and cannot be directly used for classification, we used the GCN to integrate the GrassmannCluster or HOPES algorithm for classification separately. The results showed that the MoGCN method was able to achieve state-of-the-art classification results (Figure 2). The standard deviation of MoGCN was the smallest among all compared methods, indicating that integration of the vector features and the PSN can improve the overall prediction stability. In addition, we found that using the features extracted by AE can help other classification algorithms improve their classification performance further (Supplementary Table S1). We also implemented the ablation experiments to prove that a combination of AE and SNF with GCN (MoGCN) can achieve better prediction performance. As AE and SNF are both unsupervised algorithms, the classifier method GCN is needed for subtype prediction. As shown in Figure 2, MoGCN performed better than AE + GCN and SNF + GCN in accuracy and F1 score.

### 3.3 The Interpretability of MoGCN From AE Feature Extraction and PSN Visualization

#### 3.3.1 AE Captured Cancer Gene Mutation Patterns at the CNV Level

**FIGURE 3:**
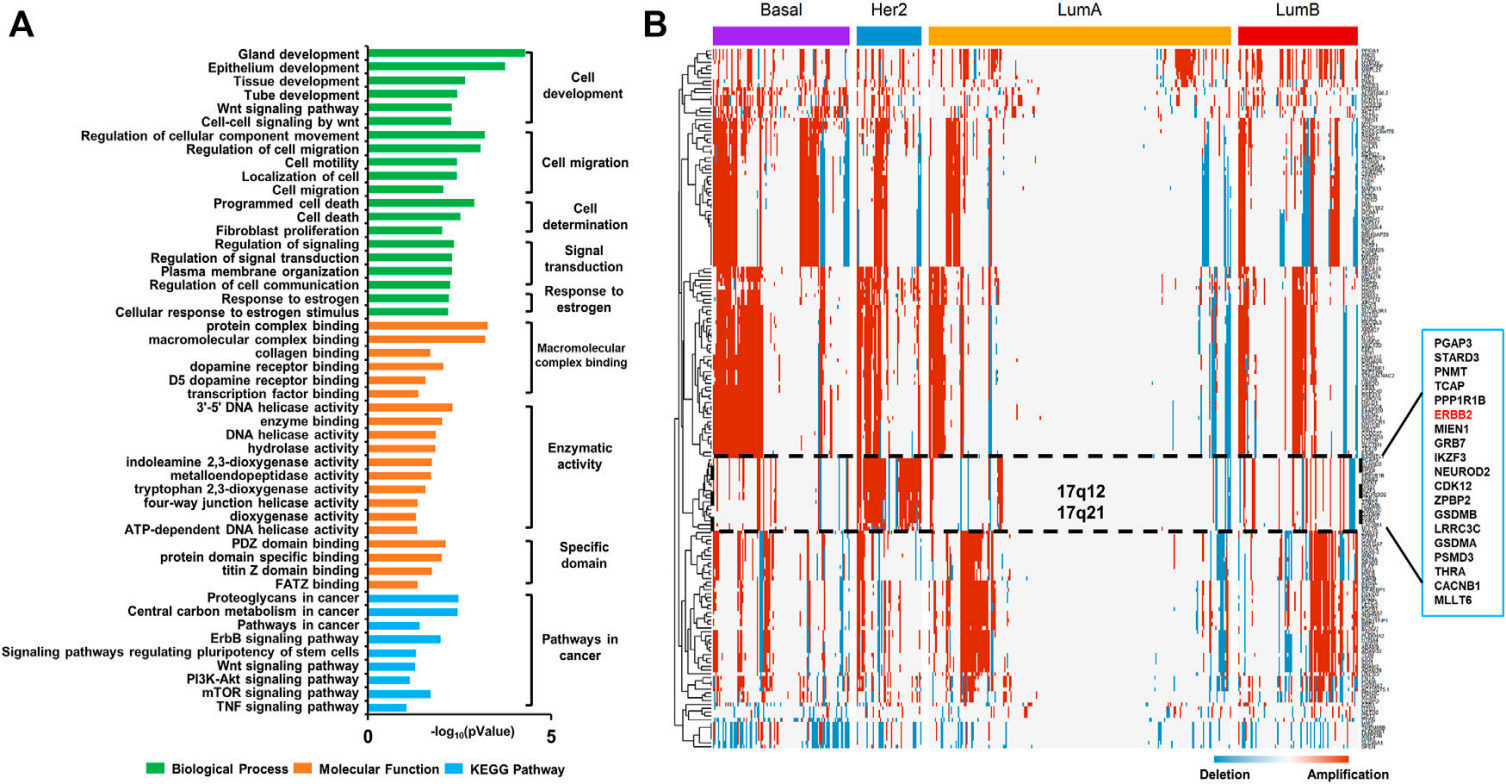
Copy number variation characteristics of breast cancer. **(A)** Biological Process (BP), Molecular Function (MF), and Kyoto Encyclopedia of Genes and Genomes (KEGG) pathway annotations for the top-scoring genes using David (*p* < 0.05). **(B)** Hierarchical clustering heat map of the mutation distribution of the top-scoring genes selected by AE.

We trained the AE for 100 epochs to converge, extracted top 100 genes with the highest scores every 10 epochs, and finally obtained 183 genes. The BP enrichment analysis of the top-scoring genes using David showed that their biological function focused on cell development, cell migration, cell death, signal transduction, and response to estrogen (Figure 3A). The KEGG annotation showed that these genes are significantly enriched in the Wnt, ErbB, PI3K-AKT-mTOR, and tumor necrosis factor (TNF) signaling pathways. The Wnt signaling pathway is highly conserved and it plays a key role in cancer progression. Mutations in the PI3K-AKT-mTOR signaling pathway are the key drivers of tumorigenesis and are related to the resistance of endocrine therapy in breast cancer. The TNF family is a group of cytokines that can cause cell apoptosis, and their expression is strongly associated with the development of various cancers. These results indicated that AE has captured genes with significant mutation patterns in BRCA.

Furthermore, we performed hierarchical clustering analysis of the selected mutation genes in all samples (Figure 3B). We found the local co-amplification of ERBB2 in the Her2-enriched subtype on 17q12-21. The role of ERBB2 as an important predictor of patient outcome and response to various therapies in breast cancer has been clearly established. It is well known that amplification of the 17q12-q21 region is the most common mechanism for ERBB2 activation in breast cancer and that it leads to the simultaneous activation of several other genes. These co-amplified and co-activated genes may have an impact on disease progression and the clinical behavior of ERBB2-positive tumors and thus represent important targets of research (Kauraniemi and Kallioniemi, 2006).

#### 3.3.2 AE Captured the EMT and Epidermal Development Characteristics of Basal-Like Subtype on Transcriptome Level

Similar as CNV data, AE selected a total of 121 candidate genes at the transcriptome level after training for 100 epochs. A cohort of 1880 patients from the KM plotter was used to validate the prognostic value of these genes. We found 70 genes that were significantly associated with 10-years overall survival (logrank *p* < 0.05), suggesting that they were potential biomarkers for BRCA prognosis (Supplementary Table S2).

**FIGURE 4:**
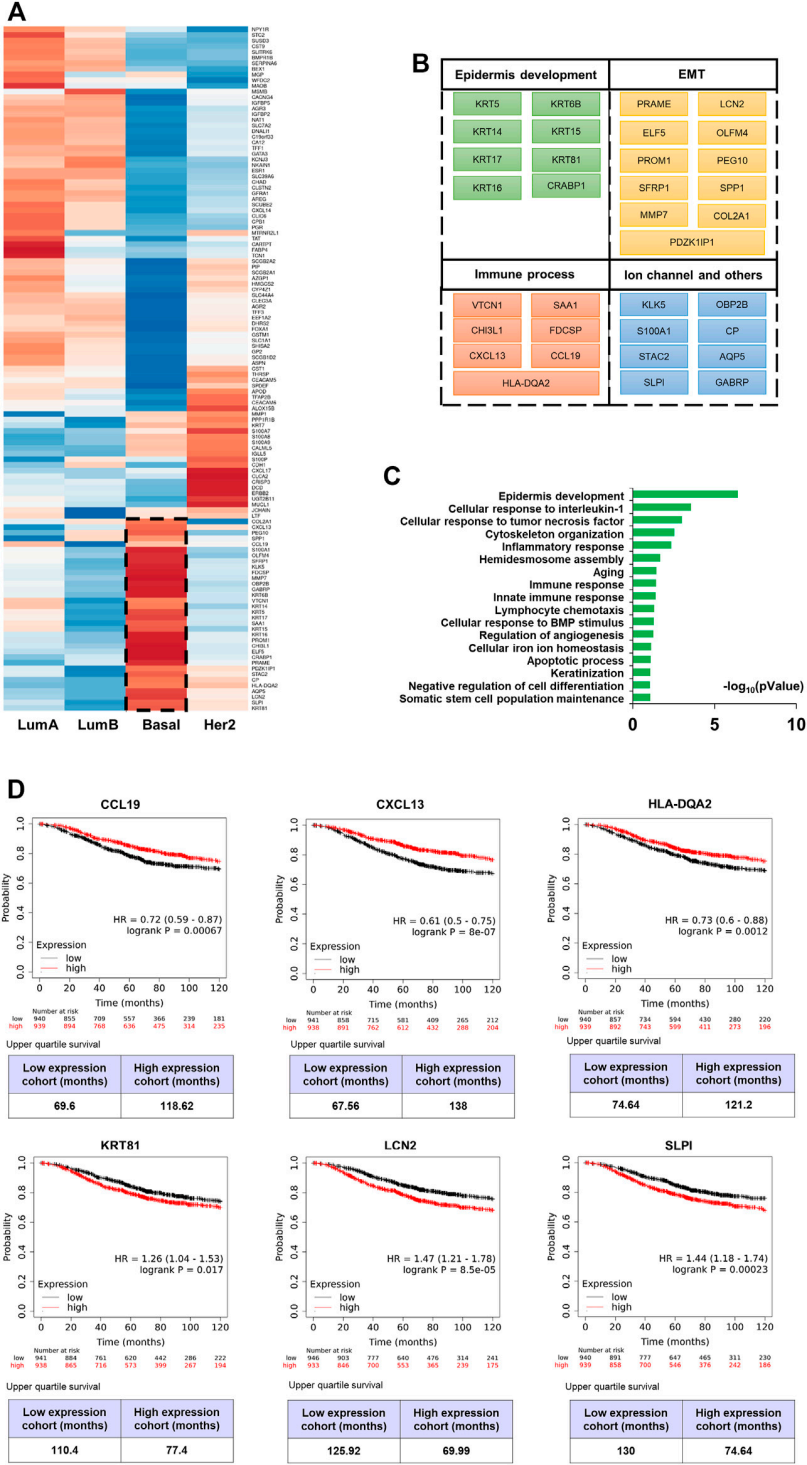
mRNA molecular characteristics of breast cancer. **(A)** Hierarchical clustering heat map of the top-scoring genes selected by AE. **(B) (C)** List of genes which are high expressed in basal-like breast cancer (BLBC) subtype and biological process (BP) annotation of these genes using David (*p* < 0.05). **(D)** 10-years overall survival analysis (logrank *p* < 0.05) of CCL19, CXCL13, HLA-DQA2, KRT81, LCN2 and SLPI.

The expression heatmap (Figure 4A) of the 121 genes was presented according to the four known subtypes (Luminal A and B, Her2-enriched, and basal-like). The BLBC patients were associated with aggressive behavior and poor prognosis, and do not typically express hormone receptors or HER-2 (the “triple-negative” phenotype). Therefore, patients with basal-like cancers are unlikely to benefit from the currently available targeted systemic therapy (Rakha et al., 2008). We focused on those genes and found that this subtype was related to epidermal development and the epithelial-to-mesenchymal transition (EMT) (Figures 4B,C). Specifically, KRT5, KRT6B, KRT14, and KRT17 are all well-described BLBC markers. KRT81 is one of the main hair proteins that is expressed in the hair cortex. However, it was reported that KRT81 is expressed in clinical specimens from patients with breast cancer (Nanashima et al., 2017). KM survival analysis showed that KRT81 is associated with poor prognosis (Figure 4D). Our results are consistent with previous studies that BLBC expresses basal cytokeratin and other markers of healthy breast myoepithelial cells. The EMT has been associated with various tumor functions, including tumor initiation, malignant progression, tumor stemness, tumor cell migration, intravasation to the blood, metastasis, and resistance to therapy. Matrix metalloproteinase (MMPs) are considered as target genes of the EMT pathway and MMP expression is a late event of the EMT (Han et al., 2018). PRAME plays a tumor-promoting role in triple-negative breast cancer by increasing cancer cell motility through EMT-gene reprogramming (Al-Khadairi et al., 2019). ELF5 is a suppressor of EMT and metastasis through the transcriptional repression of Snail2 in breast cancer (Chakrabarti et al., 2012). LCN2 modulates the degradation, allosteric events, and enzymatic activity of matrix metalloprotease-9 (Santiago-Sanchez et al., 2020). And we found LCN2 is an unfavourable prognostic factor (Figure 4D). SLPI were overexpressed preferentially in human patients that had lung-metastatic relapse (Zhang et al., 2002), its poor prognosis (Figure 4D) suggests that it may be widely related to the drivers of human cancer metastasis progression. Additionally, we found some immune factors with good prognosis, CCL19, CXCL13 and HLA-DQA2 (Figure 4D). In conclusion, these characteristics of the basal-like subtype were supported by the association between basal cytokeratins and poor outcome.

#### 3.3.3 Network Visualization and Pathway Analysis at the Proteome Level

**FIGURE 5:**
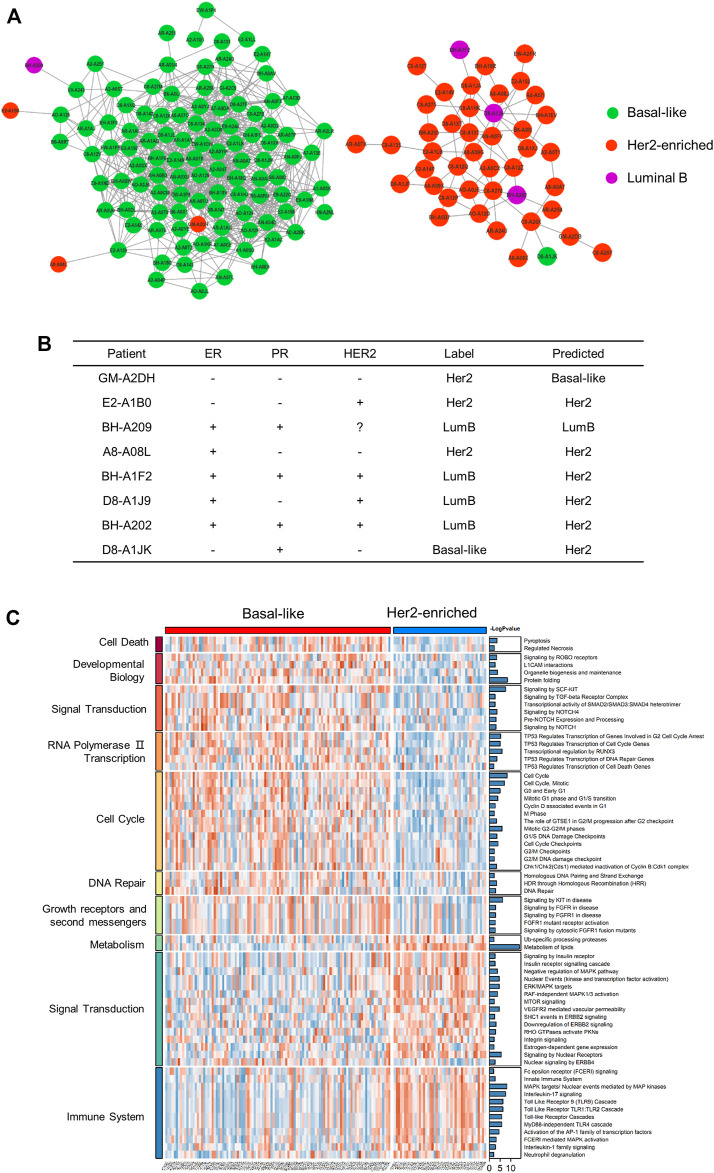
Analysis of the results for the proteome and patient similarity network. **(A)** Visualization of basal-like and Her2-enriched subgroups using Cytoscape. **(B)** The IHC, original label, and MoGCN -predicted label of patients. “−”, IHC-negative; “+”, IHC-positive; “?”, missing data. **(C)** GSVA of basal-like subgroup and Her2-enriched subgroup (*p* < 0.05).

After training the model, we reclassified the subtypes of all patients. We visualized the patient network using Cytoscape (https://cytoscape.org/) and identified the two largest subgraphs with high similarity and strong connections. These were dominated by patients with the basal-like subtype and with the Her2-enriched subtype (Figure 5A). We compared the classification results with the immunohistochemistry results (Figure 5B). In the basal-like subgroup, there were four abnormal patients (Figure 5A). Specifically, the status of GM-A2DH is ER-negative, PR-negative, HER2-negative, and it is located in the center of the basal-like subgraph. Compared with the original label Her2-enriched, it is more reasonable for MoGCN to classify GM-A2DH as basal-like subtype. Although E2-A1B0 (ER^−^, PR^−^, HER2^+^), BH-A209 (ER^+^, PR^+^, HER2^−^), and A8-A08L (ER^+^, PR^−^, HER2^−^) were connected to basal-like patients in the subgraph, their prediction results were consistent with the original labels. We suggested that this was the result of a combination of two features: 1) these patients were located at the edge of the basal-like subgraph, and 2) the multi-omics feature extracted by AE complemented the decision-making of the network. In the Her2-enriched subgroup, there were also four abnormal patients (Figure 5A), which were all predicted by MoGCN predicted as the Her2-enriched subtype. BH-A1F2, D8-A1J9, and BH-A202 were HER2^+^, indicating that they could benefit from HER2-targeting therapy. D8-A1JK (ER^−^, PR^+^, HER2^−^) did not meet the classification criteria of Her2-enriched and basal-like subtypes. Considering that it is in the Her2-enriched subgroup, MoGCN diagnosed it as Her2-enriched. These results suggested that by integrating the network structure and multi-omics features, MoGCN was able to make clinically interpretable decisions.

Considering the significant enrichment of the two subgraphs of the basal-like subtype and the Her2-enriched subtype, we performed GSVA analysis on the RPPA data of these samples (Supplementary Table S3). The results showed the statistically different pathways in two subgroups. The basal-like subgroup was more enriched in intense cell cycle activity, DNA damage repair, and the fibroblast growth factor receptors (FGFR) pathways (Figure 5C). The basal-like cell lines express an autocrine FGF2 signaling loop that may also be targetable by monoclonal antibodies (Sharpe et al., 2011), suggesting that patients harboring those tumors may be candidates for FGFR-based targeted therapies. The Her2-enriched subgroup overexpressed ErbB signaling, insulin receptor signaling, and MTOR signaling pathways, which was consistent with the genome level changes. Therefore, combination therapy targeting HER2 can effectively improve patient survival.

### 3.4 Validation of MoGCN on the TCGA Pan-Kidney Cancer Dataset

**FIGURE 6:**
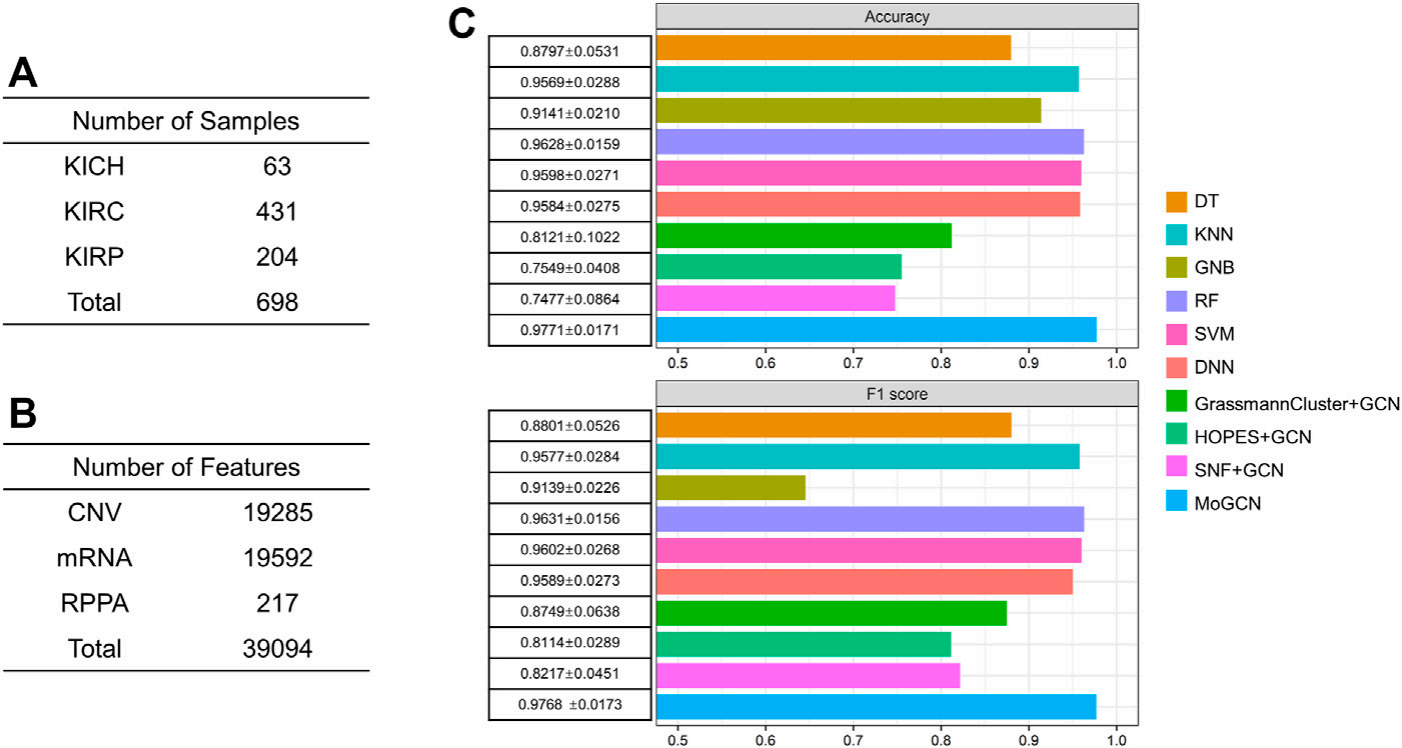
Performance of MoGCN on KIPAN dataset. **(A)**, **(B)** Summary of the KIPAN dataset. **(C)** Performance comparison of different algorithms. 10-fold cross validation (mean ± standard deviation).

To verify the generality of MoGCN, we applied this analysis model to the TCGA pan-kidney cancer (KIPAN) dataset, which consisted of three main subtypes: kidney chromophobe (KICH), kidney clear cell carcinoma (KIRC), and kidney papillary cell carcinoma (KIRP) (Figure 6A). The CNV, mRNA, and RPPA data for the 698 patients were obtained (Figure 6B). In the subtype analysis, the accuracy and F1 score of the MoGCN model reached 97.71 and 97.68% and outperformed all other compared methods (Figure 6C). These results showed that MoGCN has potential applicability for a wide range of multi-omics data mining.

## 4 Discussion

Cancer has been widely regarded as a highly heterogeneous disease, and the early diagnosis and prognostic of a cancer type have become the focus of cancer research. The ultimate goal of biology is to achieve systems biology understanding, that is, the integration, interpretation and insight of multi-omics. In the era of big data, efficient data mining of massive biomedical data is an important challenge for bioinformatics research.

We developed MoGCN, a network-based multi-omics integration pipeline for cancer subtype classification. Our study focused on the issues of feature reduction and the interpretation of prediction results. Notably, AE improved performance after integrating multi-omics features, and it also achieved the most optimal performance, which implied that it has the ability to capture the complex nonlinear relationships between multi-omics data. Whereas other mainstream algorithms slightly decreased or remained unchanged. Moreover, by using GCN to integrate the omics features and the PSN, the classification performance of our method was further improved, and displayed the highest accuracy (0.8982) and F1 score (0.9016) compared with the current mainstream cancer subtype prediction algorithms.

MoGCN is interpretative in terms of feature extraction and clinically intuitive diagnosis. Once the model has been trained, MoGCN was able to extract the most signification features of each omics layer for downstream biological knowledge discovery. The mutated genes at genome level were significantly enriched in functions or signaling pathways for cancer development, such as epidermal development, cell migration, Wnt signaling, ErbB signaling, and mTOR signaling. In addition, the genes highly expressed in the basal-like subtype with the worst clinical prognosis were characterized by enrichment in epidermal development and the epithelial-mesenchymal transition. Finally, through the visualization of the PSN, we found that the topological network and omics data features were complementary and could provide intuitive information for clinical diagnosis. The generality of MoGCN was proven on the TCGA pan-kidney cancer dataset. These case studies show that MoGCN performs well for heterogeneous data integration and the interpretability of classification results, which confers great potential for applications in biomarker identification and clinical diagnosis.

## 5 Conclusion

In conclusion, we developed an interpretable deep learning multi-omics integration model, for cancer subtype analysis. The captured features could reveal the molecular characteristics of cancer subtypes and the patient similarity network could provide intuitive information for clinical diagnosis. This study provided a novel method of the multi-omics integration. And the graph-based approach could provide new possibilities to the precision medicine.

## Data Availability

The original contributions presented in the study are included in the article/Supplementary Material, further inquiries can be directed to the corresponding authors.
